# Prostaglandin D2-Mediated DP2 and AKT Signal Regulate the Activation of Androgen Receptors in Human Dermal Papilla Cells

**DOI:** 10.3390/ijms19020556

**Published:** 2018-02-12

**Authors:** Kwan Ho Jeong, Ji Hee Jung, Jung Eun Kim, Hoon Kang

**Affiliations:** Department of Dermatology, St. Paul’s Hospital, College of Medicine, The Catholic University of Korea, Seoul 02259, Korea; jaykh86@gmail.com or kwanho16@nate.com (K.H.J.); kpajjh@naver.com (J.H.J.); mdkjeun@naver.com (J.E.K.)

**Keywords:** androgen receptor, dermal papilla cell, prostaglandin D2, AKT, CRTH2/DP2

## Abstract

Prostaglandin D2 (PGD2) and prostaglandin D2 receptor 2 (DP2) is known to be an important factor in androgenetic alopecia (AGA). However, the effect of PGD2 in human dermal papilla cells (hDPCs) is not fully understood. The function of PGD2-induced expression of the androgen receptor (AR), DP2, and AKT (protein kinase B) signal were examined by using real time-PCR (qRT-PCR), western blot analysis, immunocytochemistry (ICC), and siRNA transfection system. PGD2 stimulated AR expression and AKT signaling through DP2. PGD2 stimulated AR related factors (transforming growth factor beta 1 (TGFβ1), Creb, lymphoid enhancer binding factor 1 (LEF1), and insulin-like growth factor 1, (IGF-1)) and AKT signaling (GSK3β and Creb) on the AR expression in hDPCs. However, these factors were down-regulated by DP2 antagonist (TM30089) and AKT inhibitor (LY294002) as well as DP2 knockdown in hDPCs decreased AR expression and AKT signaling. Finally, we confirmed that PGD2 stimulates the expression of AR related target genes, and that AKT and its downstream substrates are involved in AR expression on hDPCs. Taken together, our data suggest that PGD2 promotes AR and AKT signal via DP2 in hDPCs, thus, PGD2 and DP2 signal plays a critical role in AR expression. These findings support the additional explanation for the development of AGA involving PGD2-DP2 in hDPCs.

## 1. Introduction

Androgenetic alopecia (AGA) is the most common hair loss disorder in men. AGA is characterized by the replacement of thick terminal hair with fine small vellus hair on the genetic predisposition area of scalp such as frontal and vertex area [1]. The major pathologic changes of hair follicles in AGA are hair cycle dynamics which shows gradually shortening of anagen phase. 5α-reductase plays the key role in dermal papillar cells for the transformation of testosterone (T) to dihydrotestosterone (DHT). After strong binding of DHT to androgen receptors (AR), following cascade signaling alters hair growth and affected hair follicles are miniaturized after all [2,3]. However, the mechanisms underlying AGA are not fully understood. 

Histopathologically, inflammatory cell infiltration around the follicular bulge is commonly found in AGA hair follicles [4]. Mahe et al. hypothesized that the inflammatory process in AGA is triggered by pro-inflammatory cytokines such as MCP-1, IL-6 and IL-8 [5]. These inflammatory reactions have attracted the interest of many researchers studying the underlying pathogenesis of AGA.

Cotsarelis and colleagues reported that the levels of prostaglandin D2 synthase (PTGDS) and its catalytic product, prostaglandin D2 (PGD2), were elevated in the balding scalp compared with the non-balding scalp of patients with AGA, as well as PGD2 inhibits mouse and human hair growth through DP2 [6]. The biological effects of PGD2 are usually mediated by its two G protein-coupled receptors (PGD2 receptor; PTGDR): prostaglandin receptor 1 (DP1) and prostaglandin receptor 2 (DP2, also known as chemoattractant homologous receptor expressed on Th2 cells; CRTH2). The induction of PGD2 could result from increased androgen levels, since androgens have been shown to stimulate PTGDS [7]. Some evidences suggest that PGD2 promotes the onset of catagen phase and decreased hair lengthening, leading to an increase in telogen follicles and miniaturization of the hair follicles, and PGD2 also inhibits hair follicle regeneration involved in wound healing [6,8,9]. These findings have demonstrated the effect of PGD2 on hair growth and its roles in AGA. However, our understanding of how the PGD2 pathway functions in DPCs of AGA remains limited. Thus, we focused on the expression of AR related genes by PGD2-DP2 at cellular level.

## 2. Results

### 2.1. Prostaglandin D2 Receptor 2 (DP2) Antagonist Regulates Dihydrotestosterone (DHT)-Induced Prostaglandin D2 (PGD2) Pathway

**Figure 1:**
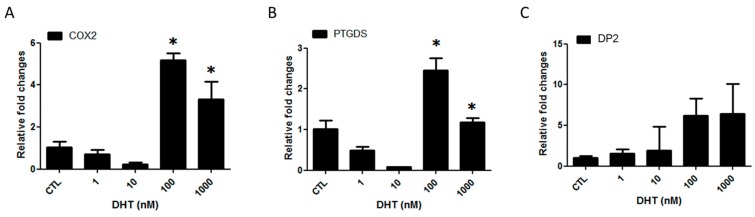

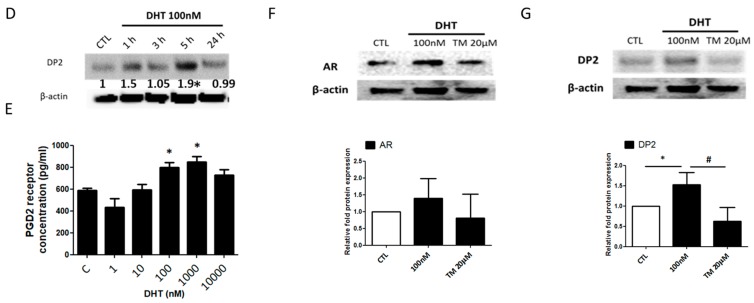
Prostaglandin D2 receptor (DP2) antagonist (TM30089) decreases dihydrotestosterone (DHT)-induced androgen receptor (AR) and prostaglandin expression in human dermal papilla cells (hDPCs). The mRNA expression of cyclooxygenase-2 (COX2), prostaglandin D2 synthase (PTGDS) and DP2 was examined in hDPCs treated with DHT for 24 h. The mRNA expression of COX2 (**A**), PTGDS (**B**) and DP2 (**C**) was induced by 100 nM DHT. DP2 protein expression was strongly induced by 100 nM DHT at 5 h (**D**). hDPCs were cultured for 24 h with DHT, as indicated. The level of PGD2 receptor in the supernatant was evaluated in three independent experiments (**E**). The relative mRNA levels were normalized to that of GAPDH. hDPCs were pretreated with 20 µM TM30089 for 1 h and then treated with 100 nM DHT for 5 h. The protein level of AR (**F**) and DP2 (**G**) was measured by western blot. TM30089 decreased the DHT-induced AR and DP2 expression. β-actin served as a loading control for protein normalization. The results are expressed as the mean  ±  SD of three independent experiments: CTL; control. * *p* < 0.05 compared with the control (0 nM DHT). # *p* < 0.05 compared with the DHT 100 nM.

To determine whether DHT affects the PGD2 pathway, human dermal papilla cells (hDPCs) were treated with various doses of DHT for 24 h. Treatment with 100 nM DHT increased cyclooxygenase-2 (COX2), PTGDS and DP2 mRNA expression (Figure 1A–C). Moreover, the protein level of DP2 was increased by 100 nM of DHT treatment at 3 h (1.4-fold) and 5 h (1.9-fold), respectively (Figure 1D). In addition, the levels of PGD2 receptor were significantly upregulated upon treatment with high concentrations of DHT, such as 100 nM and 1000 nM (Figure 1E). We next examined whether a DP2 antagonist affects AR expression. hDPCs were pretreated with TM30089 (DP2 antagonist) at 20 μM for 1 h and then treated with 100 nM DHT for 5 h. Stimulation with TM30089 inhibited the upregulation of AR and DP2 (Figure 1F,G). Additionally, immunocytochemistry data showed that DHT-stimulated AR and DP2 expression was detected in the nucleus, and the expression of AR and DP2 in the TM30089-treated group was weaker than that in the DHT-treated group (Figure S1A,B).

### 2.2. The Effects of PGD2 on AR Expression and hDPCs

**Figure 2:**
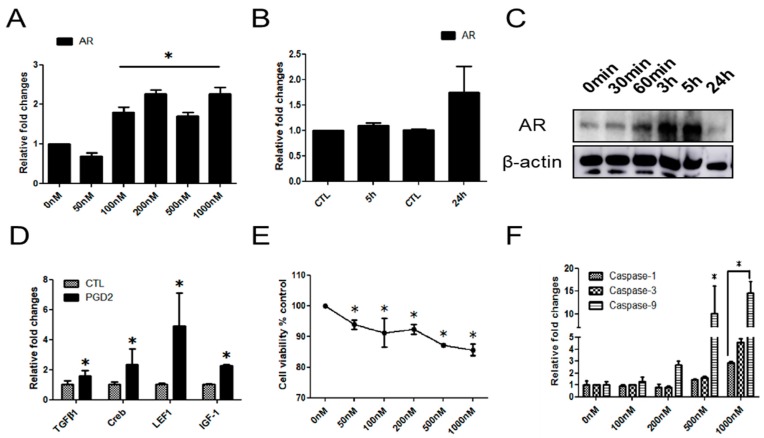
PGD2 regulates the AR expression and viability of hDPCs. hDPCs were cultured in serum-free DMEM for 24 h, and then treated with the indicated concentrations of PGD2 for 24 h (**A**). For optimal condition of AR mRNA expression, hDPCs were cultured in serum-free DMEM for 24 h, and then 200 nM PGD2 treated in hDPCs for 5 and 24 h (**B**). hDPCs were treated with PGD2 (200 nM) for the indicated times and harvested. The AR protein level was determined using western blot analysis (**C**). The mRNA expression of transforming growth factor beta 1 (TGFβ1), Creb, lymphoid enhancer binding factor 1 (LEF1) and insulin-like growth factor 1 (IGF-1) was measured by qRT-PCR (**D**). Cell viability was determined using 3-(4,5-dimethylthiazol-2-yl)-2,5-diphenyl-2-H-tetrazolium bromide (MTT) assay after incubation with different concentrations of PGD2 (0, 50, 100, 200, 500, 1000 nM) for 72 h (**E**). The mRNA expression of caspase-1, -3, and -9 was measured by qRT-PCR (**F**). The results are expressed as the mean ± SD of three independent experiments. * *p* < 0.05, compared with the control (0 nM PGD2).

To determine whether PGD2 directly regulates AR expression, hDPCs were stimulated with various concentrations of PGD2 in serum-free medium for 24 h. PGD2 at 50 nM–1000 nM induced the expression of AR. At 200 nM in particular, PGD2 treatment increased the expression of AR (2.3-fold) mRNA at 24 h compared with 0 nM group (Figure 2A). The mRNA expression of AR was increased at 24 h in compare with PGD2 treatment for 5 h group (Figure 2B). On the other hand, the protein level of AR was increased at 3 h and 5 h (Figure 2C). We examined whether AR related factors are mediated by PGD2 in hDPCs. We observed that the mRNA expression of AR related factors (TGFβ1, Creb, LEF1, and IGF-1) was increased by PGD2 treatment (200 nM for 24 h) (Figure 2D). We next examined whether PGD2 is involved in the growth inhibition of hDPCs. hDPCs were treated with various concentrations of PGD2 (0 nM–1000 nM) for 72 h. PGD2 treatment dose-dependently inhibited cell viability at 72 h (Figure 2E). Furthermore, the mRNA expression of apoptosis-related genes, including caspase-1, -3, and -9, was dose-dependently increased by PGD2 treatment for 24 h (Figure 2F). In addition, apoptosis in various concentration of PGD2 treated with hDPCs detected by TUNEL assay. We found that the number of apoptotic cells dose-dependently increased in the PGD2-treated groups (Figure S2A). Also, we examined the changes in protein levels of the Bcl2 and Bax genes, which are known to regulate apoptotic cell death. The Bax/Bcl2 ratio was 3.5-fold higher in the PGD2 (1000 nM) at 24 h compared with 5 h (Figure S2B).

### 2.3. PGD2-Induced AR Expression is Regulated by AKT Signalling

**Figure 3:**
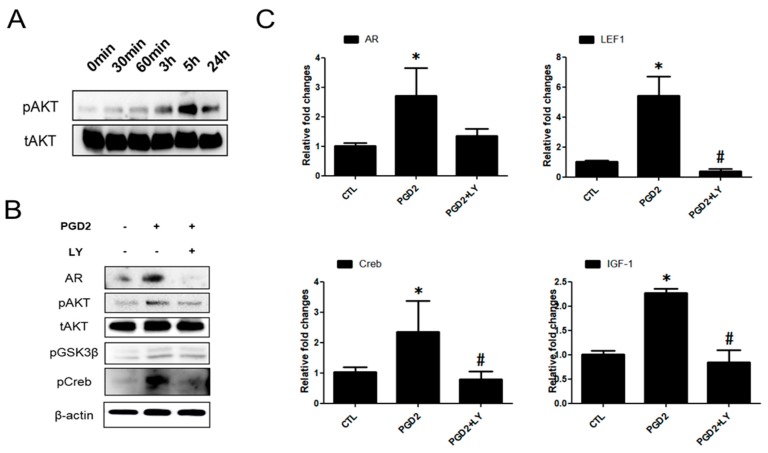
PGD2 regulates the AKT signal. The AKT phosphorylation was determined using western blot analysis. hDPCs were treated with PGD2 (200 nM) for the indicated times and harvested (**A**). hDPCs were pretreated with LY294002 for 1 h, and then PGD2 (200 nM) treatment for 5 h. The protein levels of AR, and AKT/GSK3β/Creb phosphorylation was measured using western blot analysis (**B**). The mRNA expression of AR, LEF1, Creb, and IGF-1 was measured using qRT-PCR (**C**). β-actin served as a loading control for protein normalization. GAPDH was used as an internal control for mRNA normalization. The results are expressed as the mean ± SD of three independent experiments. * *p* < 0.05, compared with the control (0 nM PGD2), # *p* < 0.05 compared with PGD2.

To investigate the association of the AKT and AR signalling pathways in PGD2-induced hDPCs, hDPCs were treated with PGD2 for different amounts of time, up to 24 h. AKT phsphorylation was observed at 3 and 5 h (Figure 3A). Second, hDPCs were treated with LY294002 (AKT inhibitor) at 20 μM for 1 h before AR, AKT and GSK3β (AKT/GSK3β) phosphorylation was analysed using western blot. Stimulation with PGD2 increased AR and AKT/GSK3β phosphorylation in hDPCs compared with control. Treatment with LY294002 along with PGD2 further decreased AKT/GSK3β phosphorylation compared with PGD2 treated group (Figure 3B). We also examined the mRNA level of AR and AKT signal related factors LEF1, Creb, and IGF-1. All mRNA of examined molecules related to the AR were blocked by treatment with LY294002 (Figure 3C).

### 2.4. PGD2-Induced AR Expression and AKT Signalling Are Regulated by a DP2 Antagonist

**Figure 4:**
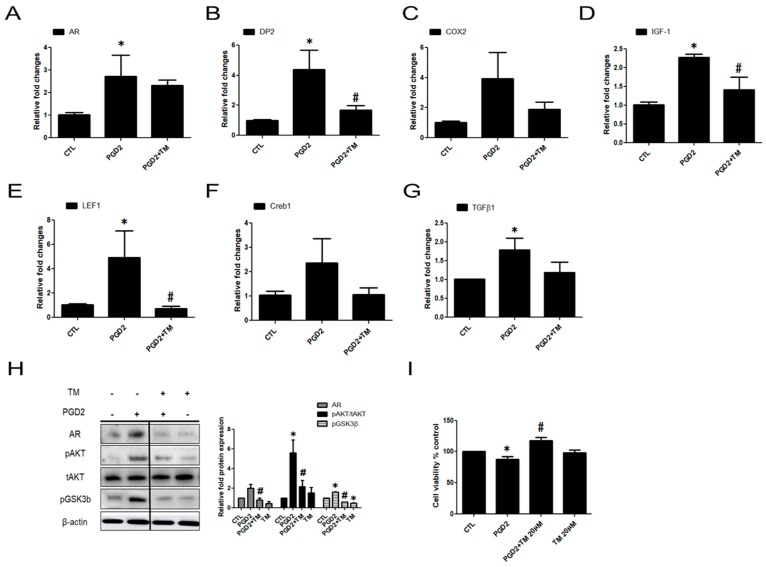
The effect of a DP2 antagonist on PGD2-induced AR related genes and AKT signalling in hDPCs. hDPCs were pretreated with TM30089 (20 µM) or LY294002 (20 µM) for 1 h and then stimulated with 200 nM PGD2 for 24 h. The mRNA expression of AR, DP2, COX2, IGF-1, LEF1, Creb, and TGFβ1 was measured by qRT-PCR (**A**–**G**). hDPCs in serum-free DMEM were pretreated with TM30089 (20 µM) for 1 h and then stimulated with 200 nM PGD2 for 5 h. The protein level of AR, and AKT/GSK3β phosphorylation was measured using western blot analysis. The histogram shows quantitative representation of the levels of PGD2-induced phosphorylation obtained from a densitometric analysis of three independent experiments (**H**). An MTT-based assay was performed to determine the effects of PGD2 after 72 h of treatment. TM30089 (20 µM) restored the viability of hDPCs that was inhibited by 200 nM PGD2 (**I**). β-actin served as a loading control for protein normalization. GAPDH was used as an internal control for mRNA normalization. The results are expressed as the mean ± SD of three independent experiments. TM; TM30089, * *p* < 0.05 compared with the control (0 nM PGD2), # *p* < 0.05 compared with PGD2.

We confirmed that PGD2-DP2 affects AR expression via AKT and its involved factors (including LEF1, Creb, and IGF-1). We hypothesized that suppression of DP2 would inactivate AR expression by inhibiting AR-related factors and AKT signalling. Thus, we examined whether inhibition of DP2 could regulate the activity of AR and its related factors. TM30089 has been known as a highly potent antagonist on mouse CRTH2/DP2 [10]. PGD2-induced AR, DP2, and COX2 mRNA expression was reduced by TM30089 (Figure 4A–C). We also found that the mRNA expression of TGFβ1, Creb, LEF1, and IGF-1, which are related to the activity of AR and AKT signalling, was blocked by TM30089 (Figure 4D–G). In addition, protein levels of AR and phosphorylation of AKT/GSK3β was also reduced by the TM30089. (Figure 4H). PGD2-inhibited cell viability was significantly recovered by 30% upon treatment with TM30089 compared with the PGD2-treated group (Figure 4I). These results indicated that AR expression and hDPC viability were regulated by PGD2 through DP2.

### 2.5. The Functions of DP2 on PGD2-Induced AR Expression

**Figure 5:**
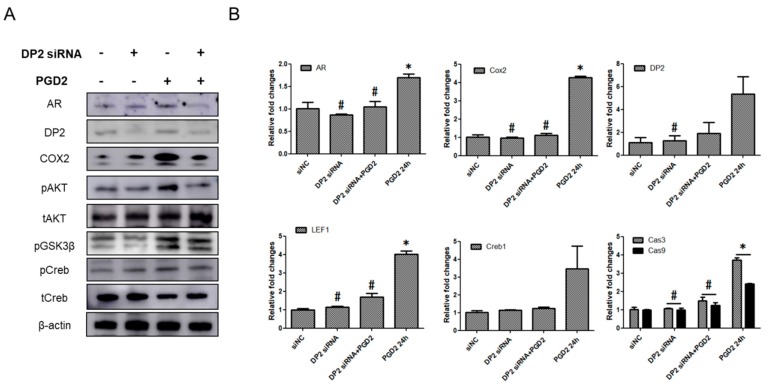
Knockdown of DP2 suppress AR related genes and AKT signal. After transfection with negative control (siNC) or DP2 siRNA, and then treated with PGD2 (200 nM) for 5 h. The protein levels of AR, DP2, COX2, and AKT/GSK3β/Creb phosphorylation was measured using western blot analysis (**A**). After transfection with siNA or DP2 siRNA for 24 h, and then treated with PGD2 (200 nM) for 24 h. The mRNA expression of AR, DP2, COX2, LEF1, Creb, and caspases (-3, and -9) was measured by qRT-PCR (**B**). β-actin served as a loading control for protein normalization. GAPDH was used as an internal control for mRNA normalization. The results are expressed as the mean ± SD of three independent experiments. * *p* < 0.05 compared with the siNC (siRNA negative control), # *p* < 0.05 compared with PGD2.

Next, to study the involvement of DP2 in PGD2-induced AR expression, hDPCs were transfected with DP2-targeting siRNA (20 nM). Transfection with DP2 siRNA significantly knocked down the protein level of AR, DP2, COX2 and AKT/GSK3β/Creb phosphorylation, whereas the negative control siRNA (siNC) (20 nM) had no effect (Figure 5A). We also confirmed DP2 gene silencing at the mRNA level. PGD2-induced the target of AR or AKT genes (including AR, COX2, DP2, LEF1, and Creb) and cell apoptosis genes such as caspase-3 and caspase-9 were markedly attenuated by DP2-targeting siRNA transfection (Figure 5B). These data suggest that DP2 is important for PGD2-mediated AKT signal on AR expression in hDPCs.

## 3. Discussion

Human dermal papilla cells (hDPCs) play an important role in hair follicle formation and hair regeneration and growth [11]. In particular, the regulation of growth and apoptosis in hDPCs has been reported to be necessary for maintaining hair growth [12]. Some studies have suggested that the factors secreted from hDPCs in response to DHT can induce male hair loss by affecting the activity of various genes in hair follicles [13,14]. DHT-induced androgens stimulate the secretion of hair growth inhibitory factors such as transforming growth factor beta 1 and 2 (TGFβ1/2) [15,16]. DHT is involved in several cellular signalling mechanisms. For example, DHT increases cell death and inhibits the cell cycle [12]. DHT modulates hair growth, hair cycling, and hair loss in AGA-susceptible hair follicles only [17]. Although definitive evidence has been reported for pathological mechanisms of AGA, the function of DPCs in AGA remain unclear.

DKK-1 and TGFβ1, which are cell death factors, are produced by DHT to destroy hair follicle cells and induce them to enter catagen stage, thereby causing hair loss [14,18]. Importantly, in susceptible individuals, DHT is also thought to precipitate an abbreviated anagen phase, as well as structural miniaturization in the hair follicle and associated anatomical structures.

Interestingly, DHT simulated prostaglandin D2 signalling through the expression of COX2, PTGDS, and DP2. Although various stimuli may induce the expression of COX2 in many cells [19,20], we used DHT, which promoted AR expression by affecting DP2 and COX2. We also investigated the changes the activity of AR by DP2 antagonist. Our results showed that DP2 antagonist has the potential to suppress AR signal by reducing the protein expression of DP2. These findings indicated that activation of AR is associated with DHT as well as prostaglandin pathway.

Cyclooxygenase-2 (COX2), a pro-inflammatory inducible enzyme, is a key enzyme in prostaglandin (PG) biosynthesis that converts arachidonic acid (AA) to PGG2 and subsequently to PGH2, which is metabolized by various PG synthases to other PGs [21]. PGs are potent biologically active lipid mediators that are produced from AA by almost every cell type and are known to regulate immune responses. One of them, PGD2 is involved in wound healing [8], and hair loss [6] actions are mediated through DP1 and CRTH2/DP2 [22]. We observed that DHT treatment enhanced the target of PGD2 pathway (COX2, PTGDS, and DP2) in hDPCs.

Based on the above results, we performed in vitro analysis to investigate the effect of PGD2 on the expression of AR in hDPCs. Firstly, we investigated the AR signal pathway involved in the effects of PGD2 in hDPCs. We focused on the TGFβ1 and TGFβ2, which involved in hair growth inhibition and apoptosis [14]. We also investigated the androgen specific transcription factors such as Creb, and LEF1, which plays an essential role in the regulation of prostate cancer cells; however, these factors have not been observed in the DPCs of hair follicles in AGA. Although IGF-1 was known as growth factor in hair development, some study reported that IGF-1 directly stimulated the activity of the 5αR and AR [23]. We found that PGD2 treatment enhances the expression of AR, Creb, TGFβ1, IGF-1, and LEF1 mRNA. Although androgens did not alter the proliferation of hDPCs [11], our results showed that PGD2 did affect the viability of hDPCs. In similar to previous findings about the effect of PGD2 on cellular viability [24,25], our results showed that at certain concentration, PGD2 can inhibit cell growth.

Apoptosis is related to the activation of caspases such as caspase-3 and caspase-9. Caspase-9, an initiator caspase, can directly cleave and activate caspase-3 [26]. We found that PGD2 treatment significantly increased the expression of caspase-1, caspase-3 and caspase-9 at 24 h. TUNEL is well known for effective method for detecting programmed cell death [27]. Our results showed that treatment with 1000 nM of PGD2 caused a significant in the number of apoptosis hDPCs. PGD2 increased the expression of *Bax* (pro-apoptotic gene), while it caused a decrease in the expression of *Bcl2* (anti-apoptotic gene) in hDPCs. These results indicate that cell apoptosis was induced by treatment with high concentration of PGD2, indicating the direct involvement of the caspase pathway.

Several studies have demonstrated that AKT is involved in signal transduction pathway downstream of a variety of inflammatory mediators, glycogen metabolism and proliferation apoptosis [28]. Phosphorylated AKT targets glycogen synthase kinase 3 (GSK3), subsequently phosphorylates GSK3β and GSK3α. Function of AKT and GSK3β were known as a key regulator of AR activation [29]. Activated GSK3β promoted the production of inflammatory molecules such as iNOS and COX2. Creb is regulated by a number of signaling kinase, including mitogen-activated protein kinase (MAPK) and AKT [30]. AKT signal that leads to induction of the AR in other cell systems [31]. Thus, we examined the role of AKT in PGD2-dependent signal pathway on AR expression in hDPCs. LY294002, a specific inhibitor of AKT, did affect the PGD2-induced upregulation of the AR, IGF-1, Creb, and LEF1 as well as phosphorylated AKT/GSK3β/Creb signal. We also observed that inhibition of AKT activation blocks the increases in expression of AR related genes induced by PGD2. Interestingly, according to previous studies, the knockdown of AKT/GSK3β suppressed AR related gene expression [32]. These results suggest that AKT/GSK3β phosphorylation is involved in AR expression. Although the role of PGD2-mediated AKT/GSK3β/Creb phosphorylation is uncertain in AR expression on hDPCs, our results indicated that PGD2-mediated AKT/GSK3β/Creb has been linked as a key factor in AR expression.

Based on the results of this study, PGD2-induced expression of AR might occur as a result of cell growth inhibition and various genes upregulation because of the activation of DP2. The DP2 antagonist TM30089 is known to regulate the viability of various cell types [10]. Thus, we observed that TM30089 has a reversal effect on the PGD2-induced decrease in cell viability. Besides, TM30089 did affect the PGD2-induced upregulation of the AR, DP2, and COX2 level as well as AKT/GSK3β/Creb phosphorylation level in hDPCs. In addition, the knockdown of DP2 (DP2 siRNA) on hDPCs did reduce the PGD2-induced AR, AKT and caspase pathway. These data indicated that AR expression of hDPCs by PGD2 was partly dependent on the presence of the DP2.

Taken together, we sought to determine which pathway(s) is critical for the induction of DP2 by PGD2 in hDPCs. Activation of DP2 by PGD2 leads to the AKT signal through G protein-dependent pathway, and more recent results show that activation of the DP2 induces apoptosis through the intrinsic pathway [33,34].

## 4. Materials and Methods

### 4.1. Human Dermal Papilla Cell (hDPCs) Culture and Reagents

Human dermal papilla cells (hDPCs, sourced from scalp of a 57-old female) were purchased from PromoCell (Heidelberg, Germany). hDPCs were cultured in Follicle Dermal Papilla Cell Growth Media (PromoCell) supplemented with provided mixture reagent at 37 °C in a humidified atmosphere of 5% CO_2_. Dihydrotestosterone (DHT) from Sigma-Aldrich (St. Louis, MO, USA). LY294002 (AKT inhibitor) were from Cell Signaling Technology (Beverly, MA, USA). TM30089 (DP2 antagonist) was from Caymen Chemical (Ann Arbor, MI, USA). For treatment, the reagents were dissolved in 100% methanol and DMSO to a concentration at 10 mM. Three to fourth-passage DPCs were used in each experiment.

### 4.2. Cell Viability Assay

hDPCs were seeded in a 24-well plate at a density of 1 × 10^4^ cells/well. To test whether DP2 antagonist participate in the viability of PGD2, hDPCs were seeded in a 24-well plate at a density of 1 × 10^4^ cells/well. After 24 h, the medium was replaced with serum-free medium. TM30089 (20 µM) were pretreated with cells for 1 h, and then incubated with or without 200 nM of PGD2 for 72 h. With the addition of 100 µL/well of 3-(4,5-dimethylthiazol-2-yl)-2,5-diphenyl-2-H-tetrazolium bromide (MTT, Sigma) to each well, the cells were incubated at 37 °C for 4 h. Then, the supernatant was harvested and then treated with 400 µL of dimethyl sulfoxide (DMSO, Sigma). The absorbance was measured at a wavelength of 570 nm using an enzyme-linked immunosorbent assay (ELISA, VersaMax Microplate, Thermo Fisher, MA, USA) reader.

### 4.3. Real Time-PCR (qRT-PCR)

Total RNA from the hDPCs using the TRIzolTM reagent (Invitrogen, Carlsbad, CA, USA) and cDNA synthesis with QuantiTect Rev. Transcription kit (Qiagen, Hilden, Germany) according to the manufacturer’s instructions. The cDNA used for real time-PCR, which was carried out with SYBR Green (Bio-Rad Laboratories, Inc., Hercules, CA, USA). The primers sequences and PCR conditions are listed in Supplementary Table S1.

### 4.4. Western Blotting Analysis

The protocol for western blot analysis was described in a previous report [35]. Briefly, the protein lysates from cultured hDPCs were prepared in RIPA cell lysis buffer containing protease inhibitor cocktail. The membranes were subsequently incubated with primary antibodies against total (AKT, Creb) and phosphorylation (AKT, Creb, GSK3β) (Cell Signaling Technology, Danvers, MA, USA), AR, COX2, Bax, Bcl-2 and β-actin (Santa Cruz Biotechnology, Inc., Santa Cruz, CA, USA), CRTH2/DP2 (Novus Biologicals LLC, Littleton, CO, USA) overnight at 4 °C on a rotary shaker.

### 4.5. Immunofluoresence of Androgen Receptor and DP2

hDPCs were plated in 8-chamber slides (SPL, Korea) at a density of 2 × 10^3^ cells per well and cultured in serum-free medium in the presence of DHT or vehicle control (methanol) for 5 h. Immunofluorescence staining of AR was performed as previously described [36]. Briefly, proteins were immunolabeled by incubating with anti-AR antibody (1:100, Cell Signaling Technology), anti-CRTH2/DP2 antibody (1:100, Novus) and anti-rabbit Alex Fluor 488 conjugated antibody (1:200; Invitrogen, OR, USA). Slides were examined under Axiovert 200 microscope (ZEISS, Germany).

### 4.6. ELISA

PGD2 receptor ELISA kit (Abbexa, Cambridge, UK) was used according to the manufacturer’s protocol. For the measurement of PGD2 receptor in conditioned medium of DHT-induced hDPCs, cells from passages 3–4 were plated overnight at a density of 2 × 10^5^ cells per 6 well culture dish, washed three times with phosphate-buffered saline (PBS), and then incubated in serum-free medium for 24 h for the collection of conditioned medium. To examine PGD2 receptor induction in response to DHT in hDPCs were treated with varying concentrations of DHT in serum-free medium for 5 or 24 h and concentrations of PGD2 receptor in conditioned medium were measured. Optical density was measured by an ELISA reader at 450 nm.

### 4.7. DP2 Gene Silencing Experiments

Small interfering RNA (siRNA) targeted at DP2 (Santa Cruz Biotechnology) was used to knockout DP2. hDPCs were cultured and incubated at 37 °C in a 5% CO_2_ incubator until 70–80% confluent. Thereafter, 2 µL DP2 siRNA duplex was diluted into 100 µL of siRNA transfection medium (Santa Cruz Biotechnology). In a separate tube, 2 µL of transfection reagent (Santa Cruz biotechnology) was diluted into 100 µL of siRNA transfection medium. The dilutions were mixed gently and incubated for 30 min at room temperature. Next, cells were incubated in negative control (siNC) or DP2 siRNA transfection cocktail for 5 h at 37 °C. Following transfection, media was changed in all cells to complete media and incubated for a further 18 h. Effects of PGD2 on AR related genes and AKT signaling in normal and DP2-silenced hDPCs were then investigated.

### 4.8. TUNEL Assay

hDPCs seeded in 24-well plates at a density of 2 × 10^4^ cell/well were treated with PGD2 at various concentrations (0, 200, 500, 1000 nM) or DMSO as a control for 72 h. Cell apoptosis was examined with the in situ cell death detection kit (Roche, Mannheim, Germany) according to the manufacturer’s instructions. The cells were fixed, permeated with 0.1% Triton X-100 solution, labelled for DNA breaks with terminal deoxynucleotide transferase (TdT) dUTP fluorescein nick end labeling (TUNEL, green fluorescence) and observed under Axiovert 200 microscope (ZEISS)

### 4.9. Statistical Analysis

All data are representative data from three independent experiments. The statistical significance of the differences among groups was tested using one-way ANOVA (SigmaPlot 12.3 software, San Jose, CA, USA). All graphs were generated using GraphPad Prism 5 (La Jolla, CA, USA). *p* value < 0.05 was considered statistically significant.

## 5. Conclusions

PGD2 directly stimulates the expression of androgen target genes, AKT and its downstream substrates are involved in mediating these effects. Thus, our data in this study provide that the activity of AR could be regulated not only DHT but also various signal changes by PGD2 in hDPCs.

## Supplementary Materials

The following are available online at http://www.mdpi.com/1422-0067/19/2/556/s1.
