# Supplementary material for: Prostaglandin D2-Mediated DP2 and AKT Signal Regulate the Activation of Androgen Receptors in Human Dermal Papilla Cells
Source: Int J Mol Sci. 2018 Feb 12;19(2):556. doi: 10.3390/ijms19020556 (PMC5855778; doi:10.3390/ijms19020556)
Supplement: Supplementary file 1 [file ijms-19-00556-s001.pdf]

Supplementary materials

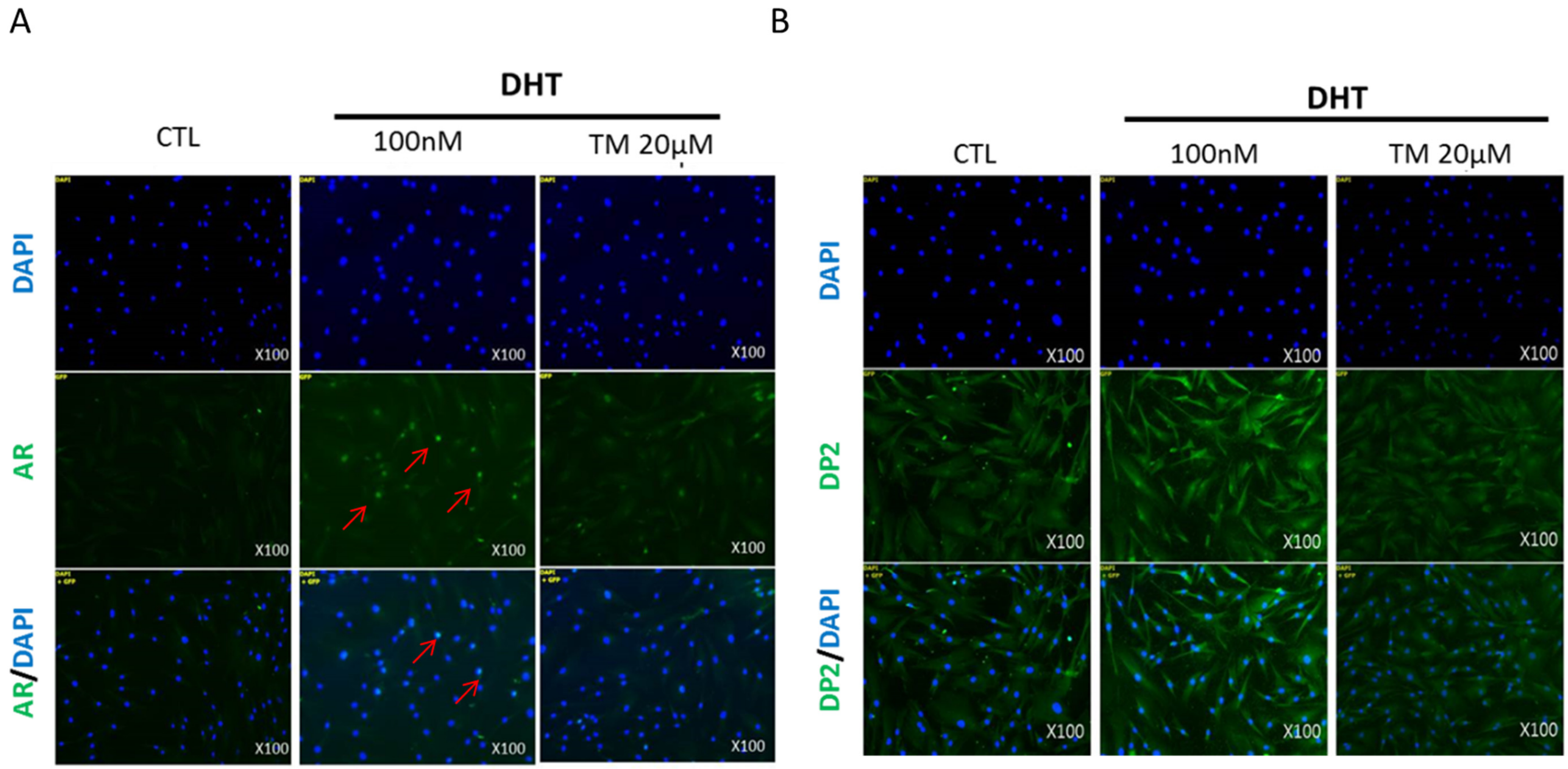

Figure S1. DP2 antagonist (TM30089) decreases DHT-induced AR and prostaglandin expression in hDPCs. hDPCs were pretreated with 20 μM TM30089 for 1 h and then treated with 100 nM DHT for 5 h. Immunocytochemical staining for AR (**A**) and DP2 (**B**) was higher in the DHT-treated group compared with cells treated with TM30089 and DHT. DAPI (blue) was used to counterstain nuclei (×100).

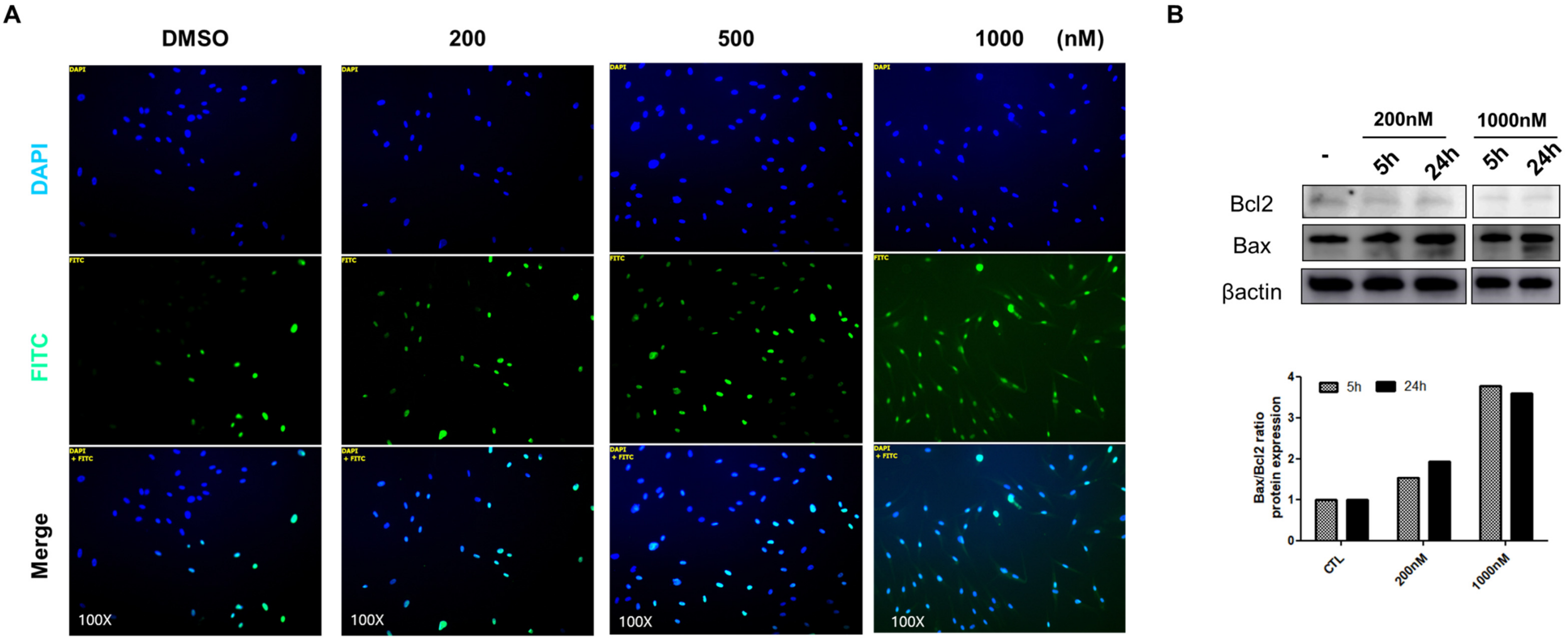

Figure S2. Effects of PGD2 on hDPC apoptotic cell death. Treatment of hDPCs with indicated concentration of PGD2. PGD2 dose-dependently increased TUNEL positive cells (**green**, FITC) compared to DMSO treated group. TUNEL positive cells in PGD2-treated groups were found to colocalize with nucleic marker DAPI (**blue**) (**A**). Bax and Bcl2 protein expression was measured by western blot assay. Protein bands of Bcl2 and Bax ratio expression (**top**) and representative graph of Bax/Bcl2 ratio (**bottom**) (**B**).

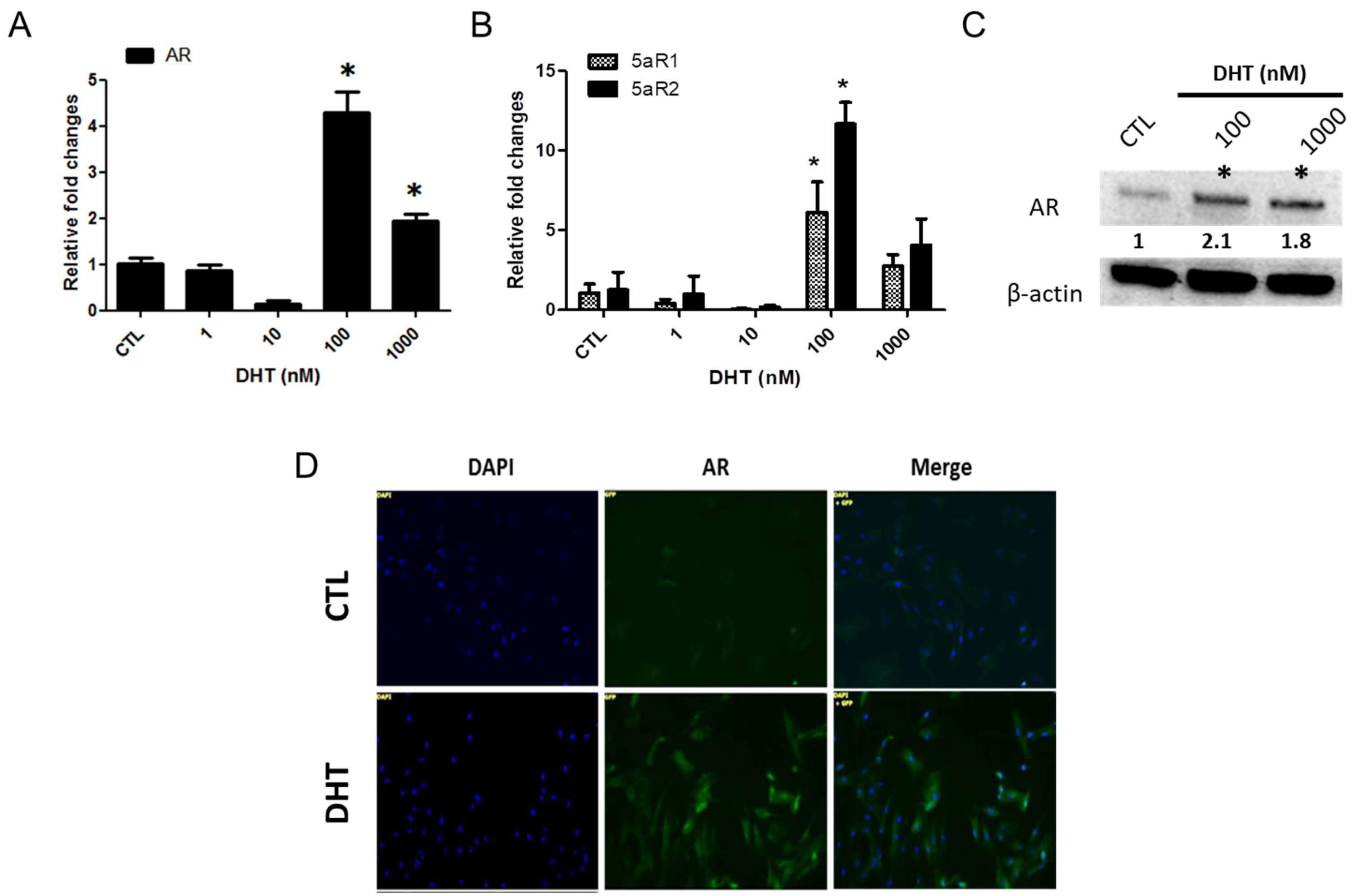

Figure S3. The effect of DHT on hDPCs. Treatment of hDPCs with DHT (100 nM for 24 h) resulted in significantly increased the androgen receptor (AR) (**A**), 5aR1, and 5aR2 mRNA expression by real time-PCR (**B**). Protein levels of AR were increased in DHT 100 nM and 1000 nM for 5 h (**C**). hDPCs were treated with DHT 100 nM for 5 h, and then AR (**green**) was strongly observed in nuclear localization by using immunocytochemistry staining (ICC). DAPI (**blue**) to counterstain the nuclei (×200) (**D**). The results expressed as mean ± S.D. of three independent experiments. CTL; control. * $p < 0.05$.

Table S1. Primers used for qRT-PCR amplications.

Primer name	Forward	Reverse	Tm(°C)
AR	GGAATTCCTGTGCATGAAA	CGAAGTTCATCAAAGAATT	51
COX2	CCCTTGGGTGTCAAAGGTAA	GCCCTCGCTTATGATCTGTC	58
PTGDS	AAAGGAGAAATTCACCGCCT	GGAGTCCTATTGTTCCGTCA	56
DP2	AGACTACAGCACACGTCATT	GTGATAGGTCAGAGGTGCAA	58
Creb	CCAGCAGCTCATGCAACATC	AGTTGAAATCTGAACTGTTTGGAC	58
LEF1	AGCTGCCTACATCTGAAACA	TGGAGACAGTCTGGGTTTTC	58
TGFβ1	CTCGCCAGAGTGGTTATCTT	AGTGTGTTATCCCTGCTGTC	58
IGF-1	AGAAGTATCAGCCCCCATCT	GTAACTCGTGCAGAGCAAAG	58
Caspase-1	TATCCGTTCCATGGGTGAAG	TCAAAGCTCGGGTCTTATCC	58
Caspase-3	GCGGTTGTAGAAGTTAATAAAGGT	CCAGGGATATTCCAGAGTCC	58
Caspase-9	TGACCCCAGAATTGACCCTG	GATTCGCTCTTGCGTCACC	58
GAPDH	GAAGGTGAAGGTCGGAGTCAA	GCTCCTGGAAGATGGTGATG	58

AR, Androgen receptor; Cox2, Cyclooxygenase2; PTGDS, prostaglandin D2 synthase; DP2, prostaglandin D2 receptor 2; Creb, cAMP response elements binding; LEF1, Lymphoid enhancer-binding factor-1; TGFβ, Transforming growth factor beta; IGF-1, Insulin growth factor-1; GAPDH, Glyceraldehydes 3-phosphate dehydrogenase
